# 

**Published:** 1819-06

**Authors:** 


### MONTHLY CATALOGUE OF MEDICAL BOOKS.

The Enemy of Empiricism, or a concise Explanation of the Functions of the Male Organs; by a genuine Disciple of Hippocrates. The second edition. 8vo. 1s.

An Essay on the Forces which circulate the Blood; being an Examination of the Difference of the Motions of Fluids in living and dead Vessels; by Charles Bell, F.R.S.E. 12mo. boards, 2s. 6d.

An Enquiry, illustrating the Nature of Tuberculated Accretions of Serous Membranes, and the Origin of Tubercles and Tumors in different Textures of the Body; with Engravings; by John Baron, M.D. 8vo. boards, 14s.

Practical Observations on the Medical Powers of the most celebrated Mineral Waters, and of the various Modes of Bathing; by Patrick Mackenzie, M.D. 12mo. boards, 4s.

An Elementary Introduction to the Knowledge of Mineralogy; by Wm. Phillips, F.L.S. &c. Second edition. 8vo. boards, 12s.

A Report of the Practice of Midwifery at the Westminster General Dispensary, during 1818; including new Classifications of Labours and Abortions, Female Complaints, and the Diseases of Women and Children; by A. B. Granville, M.D. F.R.S. F.L.S. M.R.I. &c. &c. &c. 8vo. boards, 8s.

A Treatise on Stricture of the Urethra, containing an Account of improved Methods of Treatment; with an Appendix, noticing the Application of a new Instrument to the Treatment of enlarged Prostate Gland, Gleet, Fistula, and other Diseases of the Urethra, Œsophagus, and Rectum; by James Arnott, Surgeon. 8vo. boards, 7s.

A Practical Treatise on the Efficacy of Blood-letting in the Epidemic Fever of Edinburgh; illustrated by numerous Cases and Tables, extracted from the Journals of the Queensberry-House Fever-Hospital; by Benjamin Welsh, M.D. Superintendant of that Institution, and Member of the Royal Medical Society of Edinburgh. 8vo. boards, 12s.

A Practical Treatise on the Instruction and Amusement of the Blind, calculated to promote their personal Happiness, and enable them to employ themselves with Profit and Advantage; by Doctor Guillé, chief Director and first Physician to the Imperial and Royal Institution for educating the Blind, at Paris, Member of the Institute, &c. &c. 8vo. boards, 8s.

Auxiliaries to Medicine, in four Tracts, illustrated by Engravings on Wood and Copper; by Charles Gower, M.D. 8vo. 3s. 6d.

The Hunterian Oration for the Year 1819; delivered before the Royal College of Surgeons in London, by John Abernethy, F.R.S. &c. 8vo. 2s. 6d.

On the Mechanism and Motions of the Human Foot and Leg; by John Cross, M.D. 8vo. boards, 5s.

A Letter to His Majesty's Sheriff-Deputes in Scotland, recommending the Establishment of four National Asylums for the Reception of Criminal and Pauper Lunatics; by Andrew Duncan, sen. M.D. and P. 8vo. 3s.

